# Supplementary material for: Increased chronic kidney disease development and progression in diabetic patients after appendectomy: a population-based cohort study
Source: PeerJ. 2018 Jun 13;6:e5019. doi: 10.7717/peerj.5019 (PMC6004103; doi:10.7717/peerj.5019)
Supplement: Table S1 [file peerj-06-5019-s002.docx]

S1 Table. The propensity-score model results of probability of appendectomy.

|  |  |  | **95% CI** | | ***P* value** |
| --- | --- | --- | --- | --- | --- |
| **Variables** | **Estimate** | **Odds ratio** | **Lower** | **Upper** |  |
| **Gender** | 0.1454 | 1.156 | 1.111 | 1.204 | <0.0001 |
| **age** | -0.0118 | 0.988 | 0.987 | 0.990 | <0.0001 |
| **Monthly income** | -1.84E+6 | 1.000 | 1.000 | 1.000 | 0.0052 |
| **Clinic visit frequency** | 0.0059 | 1.006 | 1.005 | 1.007 | <0.0001 |
| **Comorbidities** |  |  |  |  |  |
| **Hypertension** | 0.0588 | 1.061 | 0.974 | 1.155 | 0.1779 |
| **DM** | -0.1417 | 0.868 | 0.761 | 0.990 | 0.0346 |
| **Hyperlipidemia** | -0.0668 | 0.935 | 0.859 | 1.018 | 0.1232 |
| **CAD** | 0.0512 | 1.053 | 0.955 | 1.160 | 0.3025 |
| **CHF** | -0.1906 | 0.826 | 0.688 | 0.993 | 0.0417 |
| **Arrhythmia** | 0.1587 | 1.172 | 1.053 | 1.305 | 0.0037 |
| **Stroke** | -0.3498 | 0.705 | 0.628 | 0.792 | <0.0001 |
| **PAOD** | -0.3451 | 0.708 | 0.551 | 0.910 | 0.0071 |
| **Gout** | -0.0741 | 0.929 | 0.845 | 1.021 | 0.1239 |
| **COPD** | -0.2355 | 0.790 | 0.736 | 0.849 | <0.0001 |
| **CCI** | 0.1867 | 1.205 | 1.189 | 1.222 | <0.0001 |
| **Medications** |  |  |  |  |  |
| **Anti-diabetic** | -0.2176 | 0.804 | 0.688 | 0.941 | 0.0065 |
| **Antihypertensive** | -0.1431 | 0.867 | 0.793 | 0.947 | 0.0016 |
| **NSAIDs** | 0.0530 | 1.054 | 0.962 | 1.156 | 0.2565 |
| **Analgesic drugs other than NSAIDs** | 0.1874 | 1.206 | 1.080 | 1.346 | 0.0009 |
| **Statins** | -0.1637 | 0.849 | 0.738 | 0.977 | 0.0219 |

Abbreviations: ACEI, Angiotensin-converting-enzyme inhibitor; AF, atrial fibrillation; ARB, Angiotensin II receptor blocker; CAD, coronary artery disease; CHF, congestive heart failure; CI, confidence interval; NSAIDs, Non-steroidal anti-inflammatory drugs; PAOD, peripheral artery occlusive disease.
